# Book Reviews

**Published:** 1903-05

**Authors:** 


A MANUAL OF PRACTICAL HYGIENE. For Students, Physicians and Medical Officers. By Charles Harrington, M.D., Assistant Professor in Hygiene in the Medical School of Harvard University. Second Edition, Revised and Enlarged. Illustrated with 12 plates in colors and Monochrome and 113 Engravings. Lea Bros. & Co. Philadelphia and New York.

The suoject of hygiene is one of the highest importance to the medical profession, as well as to the public at large. The well-equipped physician should therefore be properly informed on it. This book contains all necessary information in an easily assimilable form. The subjects included are animal foods, milk and milk products, tests for contaminations, vegetable foods, beverages, air, soil, water, dwelling-houses, schools, the disposal of waste material, military and personal hygiene, disinfection. As a good guide in such matters Harrington's Hygiene will be found sufficient.

---

SURGERY OF THE HEAD. By Bayard Holmes, B.S., M.D. Professor of Surgery in the University of Illinois; Professor of Surgery in the American Medical Missionary College, Chicago, etc. New York, D. Appleton & Co. 1903.

The surgery of the head, with the exception of that of the eye, ear, nose and throat, is fully considered in the work. The chapter on cerebral localization is complete and instructive and clearly illustrated. The results of operation for microcephaly and the details of the operation are set forth. The book will be found of value as a full exposition of a subject that has hitherto been somewhat cramped by narrow position in text-books on general surgery.

---

STAMMERING AND STUTTERING, by George Andrew Lewis, and THE CULTIVATION OF THE VOICE, by Geo. B. Hynson. Illustrated. Published by G. A. Lewis, Detroit. Price $3.50.

This work is based on the assumption that this defect in speech is a mental as well as a physical disturbance. A practical scheme for cure is worked out on this theory. The sufferer, if intelligent, could go far toward curing himself by following out the teachings of this book, and to the physician it will be useful in furnishing him with the necessary guide to treating the condition.

